# Surveillance for Highly Pathogenic Avian Influenza Virus in Wild Birds during Outbreaks in Domestic Poultry, Minnesota, 2015

**DOI:** 10.3201/eid2207.152032

**Published:** 2016-07

**Authors:** Christopher S. Jennelle, Michelle Carstensen, Erik C. Hildebrand, Louis Cornicelli, Paul Wolf, Daniel A. Grear, Hon S. Ip, Kaci K. Vandalen, Larissa A. Minicucci

**Affiliations:** Minnesota Department of Natural Resources, Forest Lake, Minnesota, USA (C.S. Jennelle, M. Carstensen, E.C. Hildebrand, L. Cornicelli);; United States Department of Agriculture–Wildlife Services, St. Paul, Minnesota, USA (P. Wolf);; US Geological Survey–National Wildlife Health Center, Madison, Wisconsin, USA (D.A. Grear, H.S. Ip);; US Department of Agriculture Animal and Plant Health Inspection Service, Fort Collins, Colorado, USA (K.K. Vandalen);; University of Minnesota College of Veterinary Medicine, St. Paul (L.A. Minicucci)

**Keywords:** influenza, avian influenza, avian influenza virus, fecal sampling, H5N2, highly pathogenic avian influenza, low pathogenicity avian influenza, Minnesota, surveillance, waterfowl, wildlife disease, viruses, respiratory infections

## Abstract

In 2015, a major outbreak of highly pathogenic avian influenza virus (HPAIV) infection devastated poultry facilities in Minnesota, USA. To understand the potential role of wild birds, we tested 3,139 waterfowl fecal samples and 104 sick and dead birds during March 9–June 4, 2015. HPAIV was isolated from a Cooper’s hawk but not from waterfowl fecal samples.

Wild birds of the orders Anseriformes (ducks, geese, and swans) and Charadriiformes (gulls and shorebirds) are believed to be the predominant reservoir for avian influenza viruses (AIVs) (*1*), and most AIV subtypes are low pathogenicity (LPAIV) (*2*). Only subtypes H5 and H7 are commonly associated with highly pathogenic AIVs (HPAIVs), which sometimes arise from mutation after introduction of LPAIV in domestic poultry (*3*). The main transmission route of AIVs in birds is fecal-oral, with viral shedding in both feces and through the upper respiratory tract (*4*). Transmission involves direct or indirect contact between susceptible birds and infectious birds or fomites (*5*). A novel HPAIV (H5N2) strain discovered in North America in 2014, a reassortant with Eurasian (EA) and North American (AM) lineage genes (*6*), had been detected in domestic poultry and wild birds as far east as Kentucky, USA, through January 2016. Of 7,084 wild birds sampled by US federal and state agencies during December 2014–June 2015, a total of 98 (1.4%) tested positive for HPAIV (EA/AM H5N1, EA/AM H5N2, EA H5N8, or other EA H5); these birds were 68 dabbling ducks, 20 geese, 7 raptors, 2 passerines, and 1 diving duck (*7*).

In Minnesota, USA, HPAIV subtype H5N2 was first confirmed in a poultry facility (hereafter termed facility) in Pope County on March 4, 2015. The scope of the outbreak in Minnesota was unprecedented, and by mid-June 2015, the virus had been found in 23 counties with confirmed cases at 104 sites (98 turkey facilities, 5 chicken facilities, 1 backyard flock). The outbreak resulted in the depopulation of 9 million birds (*8*) and an economic loss of at least $650 million (*9*). Given that wild waterfowl are reservoirs for AIVs and that their movement could contribute to HPAIV spread, we conducted surveillance to detect HPAIV in wild waterfowl feces, selected dead birds, and live birds displaying neurologic impairment.

## The Study 

On March 6, 2015, we conducted an aerial survey covering a 24-km radius around the Pope County facility and identified ≈100 resident mallards (*Anas platyrhynchos*) and 21 trumpeter swans (*Cygnus buccinator*). During March 9–12, 2015, we collected 148 representative waterfowl fecal samples, pooled in groups of up to 3, to determine whether wild birds were actively shedding HPAIV. We did not detect HPAIV, although 2 pooled samples contained LPAIV (detailed methods in the Technical Appendix).

**Figure 1:**
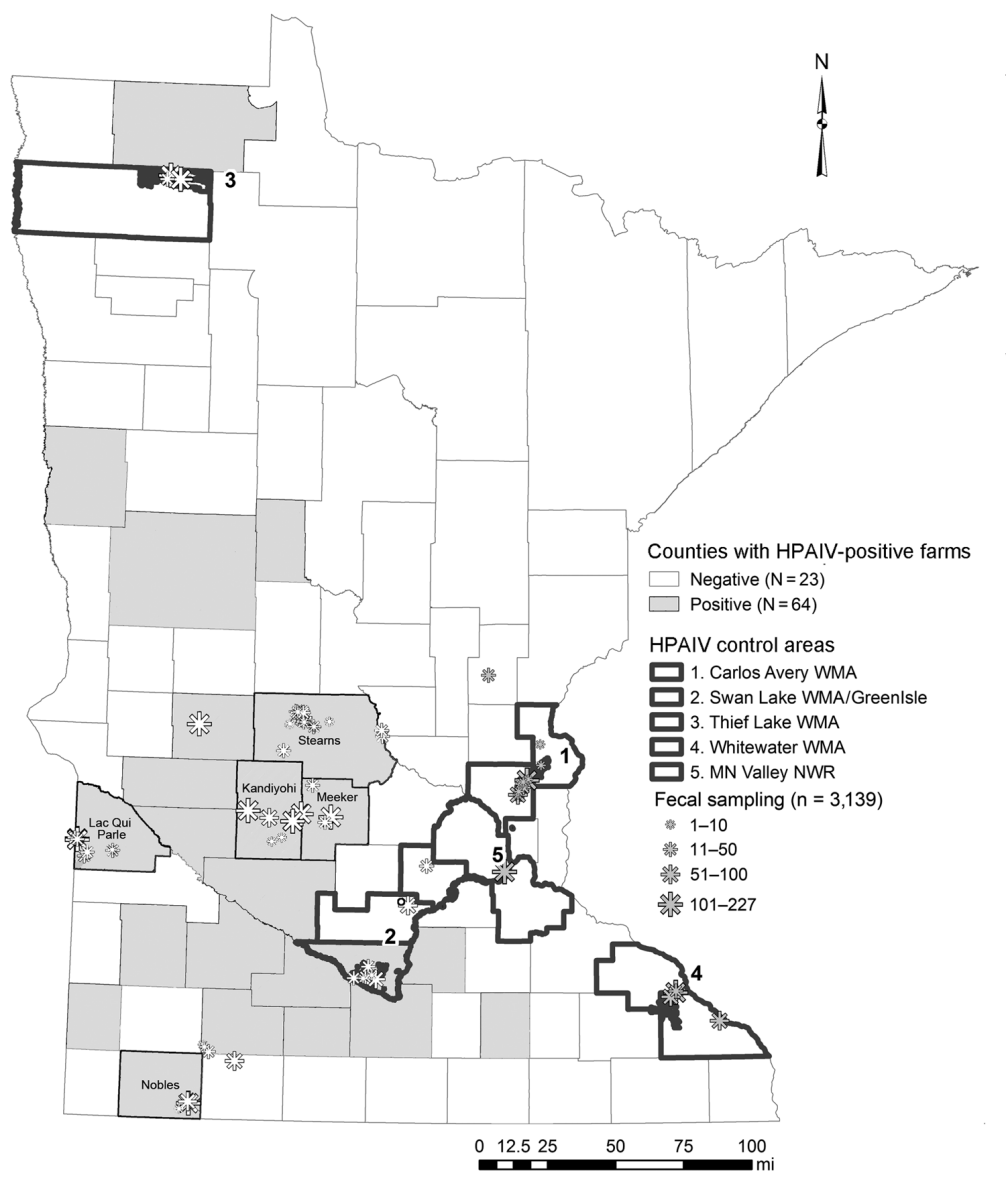
Minnesota collection sites for waterfowl feces sampled for highly pathogenic avian influenza virus (HPAIV) in spring 2015 (N = 3,139). Although HPAIV was confirmed in a Nicollet County poultry facility on May 5, 2015, our sampling occurred during April 22–April 27, 2015, and we consider this a control area (control no. 2). WMA, wildlife management area; NWR, national wildlife refuge.

In March 2015, we chose 5 counties with infected facilities (Kandiyohi, Lac Qui Parle, Meeker, Nobles, and Stearns) and 5 waterfowl production areas (Technical Appendix) where facilities were uninfected (Figure 1) to test for a spatial difference in HPAIV shedding. Within these areas, we compiled a list of wetlands and lakes and scouted those areas for waterfowl activity and sampled feces. For each area, our goal was to collect 300 fecal samples. In counties with infected poultry, we choose sites within 16 km of infected facilities. We collected ≈20 samples from a given spatiotemporal point to obtain representation within a target area.

We solicited agency staff and the public to report any deceased wild birds or live birds exhibiting neurologic signs consistent with HPAIV infection, including raptors, wild turkeys, and groups of >5 dead birds from which we obtained samples. We refer to these as morbidity and mortality samples, and our collection efforts targeted birds that had died <24 h previously.

In April 2015, which coincided with the peak rates of infection in Minnesota facilities (*8*), we collected 2,991 waterfowl fecal samples and pooled them into 1,027 brain-heart–infusion media vials; 1,591 samples (548 pooled) were obtained from counties with infected facilities, and 1,400 samples (479 pooled) were collected from waterfowl production areas without facilities (Figure 1). Although HPAIV was not detected in these samples, 30 pooled samples (representing 85 individual birds) tested positive for LPAIV. Apparent LPAIV fecal prevalence was 0.012 (95% CI 0.007–0.018) in counties with infected poultry, 0.008 (95% CI 0.004–0.014) in counties without infection, and 0.010 (95% CI 0.007–0.014) in the combined study area. Given that HPAIV was not detected and that we could not sample every individual bird in the waterfowl population, if HPAIV were present, there was a 95% probability that fecal prevalence was between 0 and 0.181% in areas with infection and 0 and 0.224% in areas without infection.

**Figure 2:**
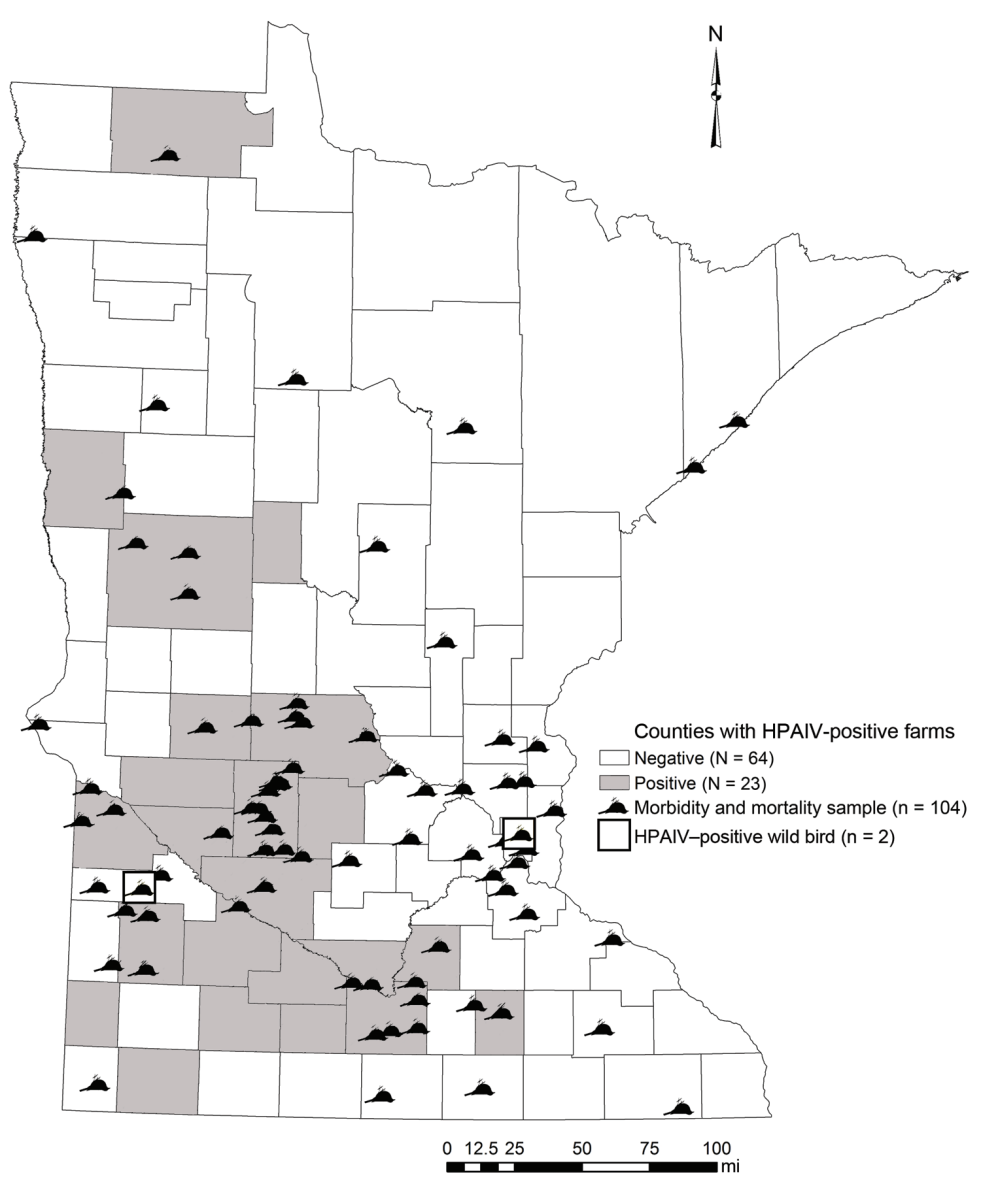
Wild bird morbidity and mortality samples (n = 104) screened for highly pathogenic avian influenza virus (HPAIV) in Minnesota through June 4, 2015. A Cooper’s hawk was confirmed to be HPAIV positive in Yellow Medicine County on April 29, 2015, whereas weak titers of Eurasian H5 RNA were detected in a sampled black-capped chickadee from Ramsey County collected in June 2015.

Through June 4, 2015 (last confirmed positive facility), we collected and tested 104 morbidity and mortality samples (Table) and detected a single HPAIV-positive bird, a Cooper’s hawk (*Accipiter cooperii*) from Yellow Medicine County (20 km from an infected facility); this infection was confirmed on April 29, 2015 (Figure 2). We suspect that this woodland predator and opportunistic scavenger was exposed to HPAIV through a food item. Although not discovered as part of Minnesota Department of Natural Resources surveillance, 3 black-capped chickadees (*Poecile atricapillus*) were found in an urban neighborhood exhibiting neurologic signs and submitted to the University of Minnesota Veterinary Diagnostic Laboratory by the Minnesota Wildlife Rehabilitation Center in June 2015; in 1 bird there was weak detection of Eurasian H5 RNA, but no virus was recovered and no sequence could be obtained directly from the sample (*7*).  All 3 birds demonstrated multifocal encephalitis, which was likely the cause for the neurologic signs (A. Armien, pers. comm.).

**Table:** Wild birds collected (n = 104) for highly pathogenic avian influenza virus screening as part of MNDNR morbidity and mortality sampling efforts, Minnesota, USA, March 9–June 4 2015

Order*	Family	Genus and species	Common name	Count
Anseriformes	Anatidae	*Branta canadensis*	Canada goose	8
		*Cygnus buccinators*	Trumpeter swan	3
		*Aix sponsa*	Wood duck	2
		*Anas platyrhynchos*	Mallard	2
Galliformes	Phasianidae	*Phasianus colchicus*	Ring-necked pheasant	8
		*Meleagris gallopavo*	Wild turkey	17
Pelicaniformes	Pelicanidae	*Pelicanus erythrorhynchos*	American white pelican	1
Accipitriformes	Cathartidae	*Cathartes aura*	Turkey vulture	1
	Accipitridae	*Haliaeetus leucocephalus*	Bald eagle	5
		*Accipiter striatus*	Sharp-shinned hawk	8
		*Accipiter cooperii*†	Cooper’s hawk	6
		*Buteo platypterus*	Broad-winged hawk	1
		*Buteo jamaicensis*	Red-tailed hawk	3
Gruiformes	Rallidae	*Rallus limicola*	Virginia rail	1
		*Porzana carolina*	Sora	1
		*Fulica americana*	American coot	9
	Gruidae	*Grus canadensis*	Sandhill crane	1
Charadriiformes	Laridae	*Larus delawarensis*	Ring-billed gull	1
		*Larus argentatus*	Herring gull	1
Columbiformes	Columbidae	*Columba livia*	Rock pigeon	2
		*Zenaida macroura*	Mourning dove	1
Strigiformes	Strigidae	*Bubo virginianus*	Great horned owl	3
Caprimulgiformes	Caprimulgidae	*Chordeiles minor*	Common nighthawk	1
Passeriformes	Sturnidae	*Sturnus vulgaris*	European starling	10
	Parulidae	*Setophaga striata*	Blackpoll warbler	1
		*Setophaga palmarum*	Palm warbler	1
	Emberizidae	*Melospiza lincolnii*	Lincoln’s sparrow	1
	Icteridae	*Euphagus carolinus*	Rusty blackbird	3
		*Quiscalus quiscula*	Common grackle	1

*1 sparrow not listed was identified to order Passeriformes. †1 HPAIV-positive Cooper’s hawk confirmed on April 29, 2015.

## Conclusions

Morbidity and mortality samples yielded the only HPAIV detected in our surveillance of Minnesota wild birds, despite the relatively small number of samples. This sample type has proven valuable for HPAIV detection in wild birds in other states; 32% of HPAIV detections nationwide and 90% of HPAIV detections within the Mississippi flyway were derived from this source during December 2014–June 2015 (*7*). Evolving HPAIV strains can elicit clinical signs and death in young immunologically naive ducks (*10*), and targeted sampling of waterfowl postbreeding areas for dead or neurologically impaired hatch-year birds might prove useful for future HPAIV surveillance (*11*).

Careful thought has been given to the design of surveillance programs for avian influenza (*12*). The study objectives, coupled with the methodologic limitations of available approaches, drive the sampling tool ultimately applied. Although opportunistic sampling (e.g., morbidity and mortality surveillance) is accessible to most agencies, it is not suited for formal population-level inferences. For estimating AIV shedding prevalence, swab sampling of oropharyngeal and cloacal cavities in live birds or the trachea and cloaca in recently deceased birds is optimal because AIV replicates and sheds through the digestive tract (*13*) and the upper respiratory system (*14*). For investigating exposure history, sampling blood from live or recently dead birds for serologic testing would be more appropriate, although timing, location, and mechanism of exposure cannot be determined.

Most of our samples were obtained from waterfowl feces. The outbreak’s speed required a quickly deployable method to collect adequate sample sizes and implement spatial design elements that would allow a meaningful comparison between known areas with infection and areas of the state apparently without infection. Modeling has shown that AIV maintenance in wild bird populations is mediated by environmental transmission (*15*), and the detection of LPAIV in waterfowl fecal samples supports that conclusion. No HPAIV was detected in waterfowl feces, although there was a 95% probability of apparent fecal prevalence throughout the study area of 0 to 0.1%. Thus, we conclude that during the 2015 HPAIV (H5N2) outbreak in Minnesota poultry, HPAIV contamination in wild waterfowl feces was not widespread. 

Technical Appendix Detailed description of methods used to detect highly pathogenic avian influenza virus in wild birds, Minnesota, USA, 2015.
