# Supplementary material for: Surveillance for Highly Pathogenic Avian Influenza Virus in Wild Birds during Outbreaks in Domestic Poultry, Minnesota, 2015
Source: Emerg Infect Dis. 2016 Jul;22(7):1278–82. doi: 10.3201/eid2207.152032 (PMC4918185; doi:10.3201/eid2207.152032)
Supplement: Technical Appendix — Detailed description of methods used to detect highly pathogenic avian influenza virus in wild birds, Minnesota, USA, 2015. [file 15-2032-Techapp-s1.pdf]

Article DOI: http://dx.doi.org/10.3201/eid2207.152032

Surveillance for Highly Pathogenic Avian Influenza Virus in Wild Birds during Outbreaks in Domestic Poultry, Minnesota, 2015

Technical Appendix

Methods

Fecal Sampling

There is a precedent established for avian influenza virus (AIV) surveillance in wild birds by sampling waterfowl feces, with comparable AIV prevalence estimates for fecal and oropharyngeal/cloacal swabbing (*1*–*5*). Given the rapid emergence of infected poultry facilities in Minnesota during April 2015, we focused on collecting waterfowl feces for the following reasons: 1) it afforded us the most control over sampling design elements and permitted hypothesis-driven surveillance (*6*), 2) a large sample size could be collected relatively quickly, and 3) the timing of the outbreak occurred when birds are not available for efficient live capture.

From March 9–March 12, 2015, we used polyester-tipped swabs to collect 148 representative waterfowl fecal samples, pooled in groups of up to 3, to determine whether ≈100 resident mallards (*Anas platyrhynchos*) and 21 trumpeter swans (*Cygnus buccinator*) were actively shedding high pathogenicity AIV (HPAIV) in the surveillance zone (≈1,830 km^2) around the Pope County index poultry facility. All fecal samples were submitted to the USDA National Wildlife Research Center (USDA-NWRC) in Fort Collins, Colorado, USA, for diagnostic testing.

For our designed sampling approach in areas of Minnesota with and without HPAIV-infected poultry, the waterfowl production areas we chose as control sites consisted of 5 wildlife management areas (WMA)/national wildlife refuges (NWR) without infected facilities (Carlos Avery WMA, Minnesota Valley NWR, Swan Lake WMA, Thief Lake WMA, and Whitewater

WMA). These areas are managed by state or federal agencies to sustain and enhance wildlife habitat (especially wild waterfowl game birds) for wildlife conservation. We used polyester-tipped swabs to collect fecal samples deposited in 1 of 17 location types (Technical Appendix Table) during April 8–April 30, 2015. We sampled what we perceived to be fresh waterfowl feces (<24 h) that were at least 2 m apart. We assumed that each feces pile represented a unique individual bird and pooled up to 3 samples per vial, which was filled with brain-heart infusion medium, and refrigerated. Samples were submitted to the USDA-NWRC in Fort Collins, Colorado, for diagnostic testing.

Morbidity and Mortality Sampling

We made no fixed goals for this sample type because of the opportunistic nature of discovery and reporting, and targeted birds that had been dead for <24 h. We used these data as an auxiliary source of information in our surveillance efforts and obtained samples statewide. Depending on the resources available for staff, we either collected whole carcasses (double-bagged and frozen) or used polyester-tipped swabs to separately obtain tracheal and cloacal specimens from sampled birds. Both swab samples from a bird were pooled in blood-heart infusion media and refrigerated. Whole carcasses were submitted to the USGS National Wildlife Health Center (USGS-NWHC) or the Minnesota Veterinary Diagnostic Laboratory in St. Paul MN, and swab samples were submitted to USDA-NWRC for diagnostic testing.

Sample Diagnostic Testing

At the USDA-NWRC, nucleic acid was extracted from 50 μL of pooled fecal swab samples using the MagMax-96 AI/ND Viral RNA Isolation Kit (ThermoFisher Scientific, Waltham, MA, USA). Five microliters of nucleic acid extracts were analyzed by real-time reverse transcription PCR (rRT-PCR) with primers and probes specifically designed to detect the influenza virus type A matrix gene (*7*) and the iTaq Universal Probes One-Step Kit (BioRad Laboratories, Hercules, CA, USA). The rRT-PCR conditions were the following: 50°C for 10 min, 95°C for 30 s, and 40 cycles at 95°C for 15 s and 60°C for 30 s. Samples with Ct values ≤38 were forwarded to the USDA National Veterinary Services Laboratory (USDA-NVSL) in Ames, Iowa, USA, for confirmation and further H5 and H7 testing and isolation.

Swabs from carcasses of diseased or dead birds were submitted to USGS-NWHC where diagnostic necropsies were performed. Tracheal and cloacal swab specimens were collected from

all carcasses and used to screen for HPAIV, when a necropsy was not performed. Tissue samples for AIV testing were homogenized in viral transport media and centrifuged at 1,000 × *g* for 30 min at 4°C. RNA from 50 µL of the supernatant of the tissue homogenate or swab material were recovered and tested for AIV by the current National Animal Health Laboratories Network protocols (*7*,*8*). Aliquots of samples for subtypes H5 and H7 were sent to USDA-NVSL for confirmation on the day of detection and were further characterized by additional tests, including rRT-PCR for virulence, sequence analysis, and virus isolation.

Data Analysis

For estimating apparent prevalence of low pathogenicity AIV) in fecal specimens, we used rRT-PCR matrix test results determined by the USDA-NVSL and applied a Bayesian approach (*9*), accounting for variable-sized pooled samples and imperfect test sensitivity and specificity. We used a binomial distribution to model the response variable (proportion of rRT-PCR positive test results) and used an uninformative prior distribution for low pathogenicity AIV prevalence. Because no published diagnostics are currently available for sensitivity and specificity of rRT-PCR matrix results from waterfowl fecal samples when specifically testing for the HPAIV(H5N2) Eurasian-American strain, we assumed unity (SE = Sp = 1). For calculating the detection threshold for HPAIV shedding in fecal samples given zero positive tests, we assumed independence among samples and used a Bayesian approach (*10*), again assuming an uninformative prior distribution beta ($\alpha = \beta = 1$) on HPAIV shedding prevalence.

References

1. VanDalen KK, Franklin AB, Mooers NL, Sullivan HJ, Shriner SA. Shedding light on avian influenza H4N6 infection in mallards: modes of transmission and implications for surveillance. PLoS ONE. 2010;5:e12851. PubMed http://dx.doi.org/10.1371/journal.pone.0012851

2. Chen H, Smith GJD, Li KS, Wang J, Fan XH, Rayner JM, et al. Establishment of multiple sublineages of H5N1 influenza virus in Asia: implications for pandemic control. Proc Natl Acad Sci U S A. 2006;103:2845–50. PubMed http://dx.doi.org/10.1073/pnas.0511120103

3. Stallknecht DE, Luttrell MP, Poulson R, Goekjian V, Niles L, Dey A, et al. Detection of avian influenza viruses from shorebirds: evaluation of surveillance and testing approaches. J Wildl Dis. 2012;48:382–93. PubMed http://dx.doi.org/10.7589/0090-3558-48.2.382

4. Ofula VO, Franklin AB, Root JJ, Sullivan HJ, Gichuki P, Makio A, et al. Detection of avian influenza viruses in wild waterbirds in the Rift valley of Kenya using fecal sampling. Vector Borne Zoonotic Dis. 2013;13:394–400. PubMed http://dx.doi.org/10.1089/vbz.2011.0926

5. Grillo VL, Arzey K, Hansbro P, Hurt A, Warner S, Bergfeld J, et al. Avian influenza in Australia: a summary of 5 years of wild bird surveillance. Aust Vet J. 2015;93:387–93. PubMed http://dx.doi.org/10.1111/avj.12379

6. Hoye BJ, Munster VJ, Nishiura H, Klaassen M, Fouchier RAM. Surveillance of wild birds for avian influenza virus. Emerg Infect Dis. 2010;16:1827–34. PubMed http://dx.doi.org/10.3201/eid1612.100589

7. Spackman E, Senne DA, Myers TJ, Bulaga LL, Garber LP, Perdue ML, et al. Development of a real-time reverse transcriptase PCR assay for type A influenza virus and the avian H5 and H7 hemagglutinin subtypes. J Clin Microbiol. 2002;40:3256–60. PubMed http://dx.doi.org/10.1128/JCM.40.9.3256-3260.2002

8. Pedersen J, Killian ML, Hines N, Senne D, Panigrahy B, Ip HS, et al. Validation of a real-time reverse transcriptase-PCR assay for the detection of H7 avian influenza virus. Avian Dis. 2010;54(Suppl):639–43. PubMed http://dx.doi.org/10.1637/8911-043009-Reg.1

9. Williams CJ, Moffitt CM. Estimation of fish and wildlife disease prevalence from imperfect diagnostic tests on pooled samples with varying pool sizes. Ecol Inform. 2010;5:273–80. http://dx.doi.org/10.1016/j.ecoinf.2010.04.003

10. Heisey DM, Jennelle CS, Russell RE, Walsh DP. Using auxiliary information to improve wildlife disease surveillance when infected animals are not detected: a Bayesian approach. PLoS ONE. 2014;9:e89843. PubMed http://dx.doi.org/10.1371/journal.pone.0089843

Technical Appendix Table. Description of 17 location types in Minnesota searched for waterfowl feces, April 2015

Location description
Foam baiting stations in ditches, ponds, and marshes
Mowed grass and gravel around ponds or along dikes
Top of gravel or grass dikes
Waste water ponds
Sand bars in lakes
Mud flats
Vegetation mats on water
Golf courses
Mowed ditches along roads
Gravel or grass shore along lakes
Upland hay meadows
Sandy beaches on lakes
Loafing rocks in open water and logs along lakes or pond shoreline
Cleared areas along lakes or ponds
Softball fields
Residence yards near ponds or lakes
Park grounds
